# Treatment fidelity monitoring, reporting and findings in a complex aphasia intervention trial: a substudy of the Very Early Rehabilitation in SpEech (VERSE) trial

**DOI:** 10.1186/s13063-022-06433-3

**Published:** 2022-06-16

**Authors:** Erin Godecke, Emily Brogan, Natalie Ciccone, Miranda L. Rose, Elizabeth Armstrong, Anne Whitworth, Fiona Ellery, Audrey Holland, Sandy Middleton, Tapan Rai, Graeme J. Hankey, Dominique Cadilhac, Julie Bernhardt

**Affiliations:** 1 School of Medical and Health Sciences, Edith Cowan University, 270 Joondalup Dve, Joondalup, Western Australia 6027 Australia; 2 Centre of Research Excellence in Aphasia Rehabilitation Recovery, Melbourne, Victoria Australia; 3 Sir Charles Gairdner Hospital, Perth, Western Australia Australia; 4 La Trobe University, Melbourne, Victoria Australia; 5 Curtin University, Perth, Western Australia Australia; 6 Stroke Division, The Florey Institute of Neuroscience and Mental Health, Melbourne, Victoria Australia; 7 University of Arizona, Tucson, Arizona USA; 8 St Vincent’s Health Network Sydney, Sydney, Australia; 9 Australian Catholic University, Sydney, Australia; 10 University of Technology Sydney, Sydney, New South Wales Australia; 11 School of Medicine and Pharmacology, The University of Western Australia, Perth, Australia; 12 Monash University, Melbourne, Victoria Australia

**Keywords:** Treatment fidelity, Behavioural therapy, Stroke, Aphasia, Rehabilitation, Randomised control trial

## Abstract

**Background:**

Treatment fidelity is inconsistently reported in aphasia research, contributing to uncertainty about the effectiveness of types of aphasia therapy following stroke. We outline the processes and outcomes of treatment fidelity monitoring in a pre-specified secondary analysis of the VERSE trial.

**Methods:**

VERSE was a 3-arm, single-blinded RCT with a 12-week primary endpoint comparing Usual Care (UC) to two higher intensity treatments: Usual Care-Plus (UC-Plus) and VERSE, a prescribed intervention. Primary outcome results were previously reported. This secondary analysis focused on treatment fidelity. Video-recorded treatment sessions in the higher intensity study arms were evaluated for treatment adherence and treatment differentiation. Treatment components were evaluated using a pre-determined fidelity checklist. Primary outcome: prescribed amount of therapy time (minutes); secondary outcomes: (i) adherence to therapy protocol (%) and (ii) treatment differentiation between control and high intensity groups.

**Results:**

Two hundred forty-six participants were randomised to Usual Care (*n*=81), Usual Care-Plus (*n*=82), and VERSE (*n*=83). One hundred thirty-five (82%) participants in higher intensity intervention arms received the minimum prescribed therapy minutes. From 10,805 (UC 7787; UC-Plus 1450; VERSE 1568) service events, 431 treatment protocol deviations were noted in 114 participants. Four hundred thirty-seven videos were evaluated. The VERSE therapists achieved over 84% adherence to key protocol elements. Higher stroke and aphasia severity, older age, and being in the UC-Plus group predicted more treatment deviations.

**Conclusions:**

We found high levels of treatment adherence and differentiation between the intervention arms, providing greater confidence interpreting our results. The comprehensive systems for intervention fidelity monitoring and reporting in this trial make an important contribution to aphasia research and, we argue, should set a new standard for future aphasia studies.

**Trial registration:**

ACTRN 12613000776707

**Supplementary Information:**

The online version contains supplementary material available at 10.1186/s13063-022-06433-3.

## Background

Clinical practice guidelines draw on the highest levels of existing evidence to provide recommendations for evidence-based practice (EBP). To evaluate a clinical intervention, treatment fidelity, in addition to effectiveness, must be considered prior to the adoption of the intervention within standard care [1] as it alters the strength and interpretation of study findings. Although the importance of treatment fidelity monitoring and reporting is recognised [2], the quality of reporting of complex interventions in clinical trials is poor [3]. This directly leads to an evidence base that struggles to guide clinical practice [3] as both intervention detail and real-world application is lacking. To address this, the Template for Intervention Description and Replication (TIDieR) checklist [3], an extension of the Consolidated Standards of Reporting Trials (CONSORT) statement [4], recommends that treatment fidelity is included as an essential interventional trial reporting component.

Treatment fidelity monitoring and reporting is critical in trial evaluations and refers to the strategies and methodological practices used to monitor the reliability and validity of behavioural interventions in a research study [5, 6]. Without the rigour that treatment fidelity brings, conclusive statements about the effectiveness of interventions cannot be made [6]. In establishing the design of an interventional study, the theoretical model underpinning the intervention should be clear and the active ingredients of the intervention should be identified prior to trial commencement. Each ingredient should be defined and subsequently monitored during the implementation of the intervention [7].

Of frameworks established to assist the application of treatment fidelity to behavioural intervention research, the Behavioural Change Consortium (BCC) [5] is the most widely adopted [8]. The BCC outlined treatment fidelity monitoring and reporting recommendations designed to link the theory of a complex behavioural intervention to the application of that intervention. The five areas identified by the BCC were (1) study design, i.e. strategies to test theory driven hypotheses; (2) training providers, i.e. methods used to ensure therapists are adequately trained to deliver the intervention; (3) delivery of treatment, i.e. strategies to determine if the treatment is delivered as planned; (4) receipt of treatment, i.e. ability of patients to demonstrate during the intervention that they understand and can perform the behavioural skills in the intervention; and (5) enactment of treatment skills, i.e. demonstration by the participants of the ability to use the intervention in real life settings.

In a recent review of 93 studies, less than 30% reported actual or planned fidelity in aphasia treatment studies with information on where the treatment was provided, individual treatment tailoring, and modification rarely reported [9]. Reviews of treatment fidelity processes confirm limited treatment fidelity reporting in aphasia treatment studies [10–12]. Authors of more recent aphasia trials have published standalone treatment fidelity protocols [13–16] reflecting an increased focus on reporting treatment fidelity in aphasia research and incorporation of more rigorous procedures in study design and evaluation.

### Aim

We aim to outline the treatment fidelity monitoring processes and results of the large, multi-centre VERSE trial.

## Methods

### Background to the VERSE trial

VERSE [17] was a three-armed RCT consisting of UC, UC-Plus and ‘VERSE’ aphasia therapy with therapy provided as follows:

- UC therapy was provided by a qualified speech pathologist, a qualified therapy assistant or a speech pathology student, being directly supervised by a qualified speech pathologist. Aphasia treatment was delivered on a 1:1 basis or in small groups.
- UC-Plus therapy was defined as per UC with the additional intensity requirement of 20 sessions of 45–60 min (15–20 h of direct aphasia therapy within a maximum of 50 days post stroke. This allowed an extra 7 days to complete treatment to account for illness or other factors).
- VERSE was a prescribed intervention for treatment type and provided at the same intensity as UC-Plus.

UC and UC-Plus sessions were used as the “active concurrent control” for treatment intensity (H1) and type (H2) comparisons in the trial. A full description of therapy including setting, participant inclusion and exclusion criteria, intervention description and planned data analysis is provided in the VERSE trial manual in the main trial publication supplement [17]. Differences in the type of treatment provided in UC and UC-Plus were not controlled for unless the intervention was deemed outside the above description. The results of the primary analysis showed that early, intensive aphasia therapy did not improve recovery of communication, as measured by the Western Aphasia Battery-Revised Aphasia Quotient (AQ) at 12 weeks after stroke [17].

Treatment fidelity adherence monitoring for the three arms of the trial was documented for the prescribed study phases as per TIDieR [3]. Assessment fidelity is reported in the supplement. UC was monitored only for compliance to the main study protocol which was necessary to maintain the control arm of the trial. Further monitoring of the control arm was not undertaken to avoid a Hawthorne effect bias, or change in behaviour due to being watched [18]. Therefore, this paper contains reporting and comparisons only for the UC-Plus and VERSE arms in the trial. The VERSE treatment fidelity analysis focused on two key areas that aligned to the primary and secondary hypotheses of the trial:

1. Primary: the amount of therapy provided to participants in the intensive arms to confirm the prescribed amount of therapy met protocol requirements
2. Secondary: the nature of the therapy provided to verify that VERSE therapy was adherent to the protocol and different to the UC arms of the study.

### Data extraction

Data were extracted from the VERSE trial’s data management system (REDCap® [19]) containing therapy session logs, protocol deviations and therapist and participant details. A secured electronic data transfer system compliant with international data safety requirements (Cloudstor^TM^) was used for the transfer of video-recorded sessions. The trial data monitor downloaded the files to a central database, monitored by dedicated security systems.

### Treatment fidelity procedures

An independent treatment fidelity monitor was employed to complete compliance checks related to commencement of treatment (on or before day 15 post stroke) and that the intervention period did not exceed 50 days post stroke. The treatment fidelity monitor was responsible for providing feedback to all therapists (UC, UC-Plus and VERSE), about any deviations from the therapy protocol and responded to general questions about the treatment and assessment procedures. They also reviewed the recorded treatment sessions to determine if the prescribed VERSE protocol was adhered to. A treatment fidelity co-ordinator supervised this process and provided specific feedback to VERSE therapists in the event of reduced compliance reported by the treatment fidelity monitor. A database that recorded adherence and differentiation of data was maintained. An independent research assistant cross checked and summarised these data. Trial investigators were blinded to the fidelity processes and results during the trial and received only summary data to ensure the trial was progressing as prescribed. The treatment fidelity co-ordinator whose role was to oversee the treatment integrity processes was the exception to this.

Table 1 details the way treatment fidelity was conceptualised in the study design phase as per TIDieR Item 11 ‘How well planned’ [3] and the BCC recommendations [5]. TIDieR Item 12 ‘Actual’ [3] is addressed in the ‘Results’ section of this paper. In line with recommendations, the theoretical underpinnings of the intervention were considered and specified to allow monitoring. The prescribed VERSE intervention was founded on principles of promoting neuroplasticity through targeted early intensive language therapy based on the patient’s impairment(s). The main principles that guided the VERSE prescribed intervention were (i) massed practice, (ii) error-free learning, (iii) task complexity, (iv) salience and (v) maximising communicative success through interactive functional tasks. Adherence to these components was monitored using the therapy integrity monitoring form (Supplement 1).

**Table 1:** VERSE reporting for TIDieR and Behaviour Change Consortium treatment fidelity recommendations

Area*	Details*	Application to VERSE trial
Study design	Ensure same treatment dose within conditions	• The intervention time for the VERSE trial was a maximum of 25 working days after baseline assessment; allowing up to 14 days to recruit and assess, the intervention period for all participants ceased at day-50 post stroke if not completed earlier. • The two intensive arms were designed to deliver the same dose within each condition (20 sessions of 45–60 min or 15–20 h of direct aphasia therapy). Each group was monitored to confirm therapy amount was within the prescribed range.
	Ensure equivalent dose across conditions	• All video-recorded sessions were cross-checked with data entered into REDCap® [19] to ratify correct number and length of sessions, treatment type (direct aphasia therapy as compared to assessment, counselling or education) and allocated treatment group (e.g. VERSE treatment as compared to Usual Care therapy). • Usual care therapy dose was expected to vary within the condition as per the control design.
	Plan for implementation setbacks	• An additional five working days (to maximum day-50) was allowed for the intensive intervention to be completed due to known treatment barriers in early stroke recovery. • A pool of ‘intensive therapists’ trained exclusively in each intensive regimen, i.e. UC-Plus and VERSE arms, was available so that implementation did not rely on one therapist. • Provider attrition was tracked through a VERSE substudy.
Training providers	Standardise training	• All assessors and therapists in intensive regimens were qualified speech pathologists. • All therapists and assessors received 1–3 h of face-to-face or videoconference training. • Usual Care, UC-Plus therapists, Principal Investigators, baseline and blinded assessors received (relevant to their role): o Written study protocol o Training power point slides o Written therapy and data entry manuals o Access to a ‘training mode’ REDCap® [19] database o Introduction and access to trial monitoring staff for support throughout the trial o Assessment kits (standardised assessments, video recorder, recording forms) • VERSE therapists received the above materials *and*: o Specific additional VERSE training emphasising the prescribed treatment regimen o VERSE-specific training manual o VERSE treatment plans for each goal of the treatment hierarchy o Pre-reading material outlining treatment theory o VERSE treatment task hierarchy o Therapy recording sheets o Video-recorded examples of VERSE therapy o Frequently asked questions document o Standardised VERSE training kit/therapy materials
	Ensure provider skill acquisition	• VERSE therapists were encouraged to ‘practise’ the treatment regimen before commencing trial treatment. • VERSE and UC-Plus therapists were required to video-record one therapy session per week (approx. four recordings per participant). For practical reasons therapists were encouraged to record every 5th session however, therapy videos for any session were accepted. Video recording for therapists providing usual care sessions was as per standard care. • Baseline and blinded assessors’ audio recorded connected speech samples as part of the assessments. Feedback from the treatment fidelity monitor was provided if the assessor required support in elicitation of either monologic or dialogic samples.
	Minimise therapist drift	• Monitoring of therapy videos per above. • Ongoing updates and reminders about the trial were included in monthly VERSE newsletter which included generic treatment tips. • UC and UC-Plus therapists were encouraged to contact the treatment fidelity monitor with treatment queries. • VERSE therapists contacted the treatment fidelity co-ordinator with treatment queries. • Videos were examined as soon as received. General session feedback (e.g. length, frequency of session) for VERSE therapists was provided by the treatment fidelity monitor. Treatment-specific feedback was provided by the treatment fidelity coordinator if 20% of the targeted therapeutic interactions / behaviour was deemed non-adherent (Fig. 1).
	Accommodate provider differences	• The VERSE Expert Advisory Committee determined that 80% was a clinically acceptable level of protocol adherence whilst accommodating for provider differences within sessions. A session was considered compliant if 80% of the total interactions and activities within the session were adherent.
Delivery of treatment	Control for provider differences	• All video-recorded sessions were reviewed and rated against key criteria (Supplement 1). Non-compliant sessions were defined as sessions containing more than 20% of non-compliant interactions. If UC-Plus sessions did not comply with treatment frequency and session length, the treatment fidelity monitor raised the issue with the treating therapist. If VERSE treatment sessions were deemed non-compliant by the treatment fidelity monitor, they were escalated to the treatment fidelity coordinator who addressed the non-compliant behaviour with the therapist. • Regular communication to VERSE therapists to remind them of key therapy features
	Reduce differences within treatment	• Written therapy manual and video-recorded examples of treatment at different difficulty levels were provided to therapists. • Feedback provided to therapists if main and intervention protocol adherence needed to be addressed. • Ongoing updates and reminders about the trial were included in a monthly VERSE newsletter.
	Ensure adherence to protocol	• Videoed therapy sessions were monitored for protocol adherence per above. • Example VERSE therapy plans with scripted task explanations were given to therapists • Therapy deviations were reported and recorded in REDCap® [19]. • Ongoing regular access to trial staff for questions and direction as required.
	Minimise contamination between conditions	• Treatment materials were specific to the intervention arm of the trial, marked confidential and only provided to therapists within that arm. • VERSE therapists were trained specifically to minimise intervention contamination and were instructed not to disclose VERSE therapy to non-VERSE therapists or assessors. • Once trained, VERSE therapists could not undertake a UC or UC-Plus role. • Sessions were conducted in a quiet and private place so as not be in ear-shot of others. • Videoed sessions were reviewed to identify any contamination between the arms of the trial.
Receipt of treatment	Ensure participant comprehension	• Aphasia-friendly informed consent was obtained. • Participants with very low comprehension recruited to the trial were supported in the intervention as required by trained Speech Pathologists. • VERSE arm participants were treated with an intervention plan that stipulated a demonstrated comprehension of at least 50 single words (Goal 1 was a comprehension only goal) before progressing to Goal 2. See supplementary materials supplement for treatment goals.
	Ensure participant ability to use cognitive skills	• The intervention was structured around achievement-based objectives • VERSE therapy included the introduction of incremental levels of communication complexity involving comprehension and verbal expression. If 80% accuracy on the current goal was achieved, it was determined that the participant had sufficient comprehension and cognitive skills to progress to the next therapy goal
	Ensure participants ability to perform behavioural skills	• Success at each level within the VERSE treatment programme meant the participant had sufficient comprehension and cognitive skills to perform the desired behaviours. All 20 VERSE therapy sessions and goals were recorded, providing a clear outline of the development of targeted behaviours
Enactment of treatment skills	Ensure participant use of cognitive skills	• Enactment of these skills beyond the therapy session was not monitored and not a focus of this research.
	Ensure participant use of behavioural skills	• Per above.

*TIDieR [1] Item 11. How well planned; ‘Area’ and ‘Details’* columns from Behaviour Change Consortium treatment fidelity recommendations [2]

## Results

TIDieR Item 12 ‘Actual’ stipulates the monitoring and evaluation of intended treatment fidelity aspects as they eventuated in the trial [3]. We have conceptualised this as the results of the planned treatment fidelity procedures reported in the ‘Methods’ section. Results are presented as per the Study Design, Training Providers and Delivery of Treatment areas of the Behaviour Change Consortium recommendations [5]. The receipt of treatment and enhancement of treatment skills areas of the recommendations were planned into the study as per Table 1 TIDieR Item 11 however, not monitored further due to constraints of the trial design.

### Study design

A total of 10,805 speech pathology sessions (UC 7787; UC-Plus 1450; VERSE 1568) were recorded. Reported UC services include all services for communication and swallowing, assessment and treatment for all groups. UC-Plus and VERSE sessions were for aphasia treatment only, and these form the basis for the analysis related to ensuring a difference between therapy types.

Within the UC-Plus arm, 61 of 81 (75%) participants received the minimum intensity of greater than 15 h of intervention, while, within the VERSE intervention, 72 of 83 (86%) received this dose.

**Fig. 1:**
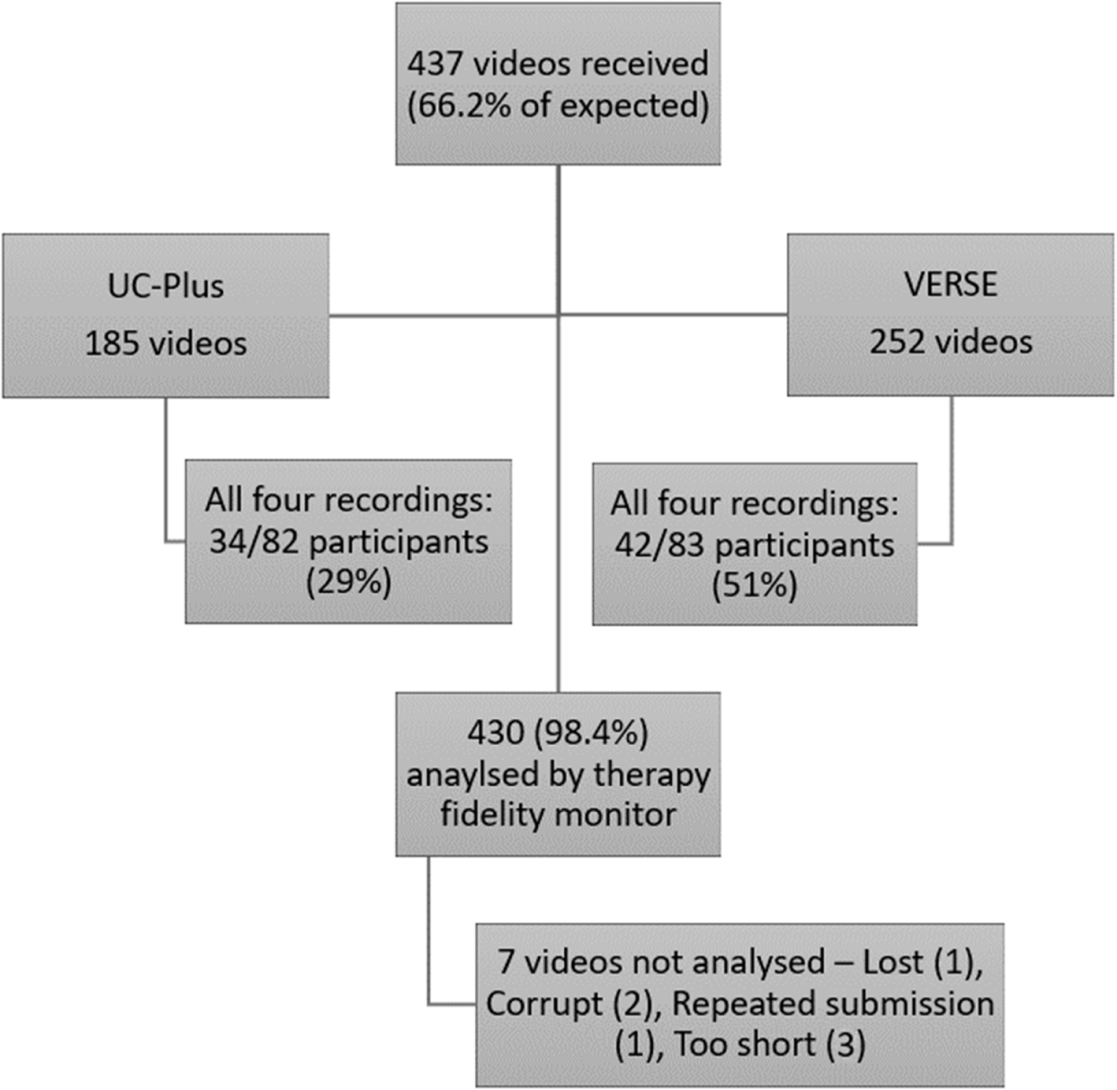
Videos received in the trial

Based on the number of participants per intensive intervention arm (UC-Plus *n*=82; VERSE *n*=83) and the recommendation that each therapist submit four videos over the therapy period, 660 videos were planned while 437 were received. Two videos were received from the UC arm of the trial where video recording was not mandated and have not been included in this analysis. Figure 1 describes videos received and analysed and outlines the reasons for non-analysis.

### Training providers

Therapy was delivered by 430 therapists in total (UC 200, UC-Plus 142, VERSE 98) across 17 acute care and 45 subacute participating hospital sites. Some therapists worked across multiple sites. Therapists were predominantly female (*n*=418, 97.2%). In the higher intensity arms of the trial, 100% of therapists received systematised training (2–3 h) and training materials (manuals, therapy materials, recording sheets). Therapists in the VERSE arm received additional VERSE-specific training (2 h). Quarterly meetings were held for UC-Plus and VERSE therapists for the duration of the intervention. To encourage recruitment and adherence to protocol, 43 newsletters and 24 mid-monthly updates were sent to therapists from 15 July 2014 to 30 April 2018.

### Delivery of treatment

A standard therapy protocol was written and disseminated to therapists. Two complaints were received from participants during the trial regarding their therapist. Six potential treatment contamination deviations were logged in the VERSE treatment arm (a VERSE trained therapist completed usual care therapy with a study participant as part of their routine employment). However, the therapists’ treatment deviations were reported as *not using* the VERSE treatment protocol to treat language but instead providing Usual Care for dysarthria and or apraxia of speech. No other reports of contamination were received.

From 10,805 total intervention episodes in the trial, 3077 (28.5%) were related directly to aphasia intervention in the UC-plus (1505) and VERSE (1572) groups during the intervention period. Of these treatment sessions, 431 therapy deviations were reported. There were 36% more deviations reported in the UC-plus group compared to the VERSE group. The quantity and reasoning are provided in Table 2.

**Table 2:** Therapy deviations recorded from REDCap logged deviation forms

	UC-Plus (*n*=294) *n* (%)	VERSE (*n*=137) *n* (%)
Did not receive minimum number of sessions *per week* Reasons	41 (13.9%)	24 (17.5%)
Deceased	0 (0%)	1 (0.7%)
Medically unwell	5 (1.7%)	4 (2.9%)
Participant off ward	3 (1%)	4 (2.9%)
Refused intervention	17 (5.8%)	6 (4.4%)
Therapist not available	5 (1.7%)	2 (1.5%)
Fatigue	1 (0.3%)	1 (0.7%)
Other^a^	10 (3.4%)	6 (4.4%)
Deviation in therapy *session length* Reasons:	253 (86.1%)	113 (82.5%)
Fatigue	62 (21.1%)	23 (16.8%)
Medically unwell	34 (11.6%)	22 (16.1%)
Participant declined/refused	84 (28.6%)	9 (6.6%)
Other^a^	73 (24.8%)	59 (43.1%)

^a^Reasons given were highly varied

Therapy deviations were recorded for 114 participants across all sites. Multiple regression was used to determine if baseline patient and or stroke factors influenced the likelihood of having a therapy deviation. Results showed that 10% of the variance in reported therapy deviations can be collectively accounted for by five factors, *F*(5,432)=9.83), *p*=.000. Age (*β*=.282, *t*=2.377, *p*=.018), lower AQ score (*β*=−.351, *t*=−4.66, *p*=.000) and being in the UC-Plus group (*β*=−8.883, *t*=−2.749, *p*=0.006) positively predicted treatment deviations. A higher stroke severity score (NIHSS) (*β*=−1.582, *t*=−5.726, *p*=0.000) predicted more treatment deviations.

### Therapy adherence and differentiation

Videoed therapy sessions in the VERSE arm were marked as adherent or non-adherent by the treatment fidelity monitor according to key therapeutic elements. Sessional adherence data is in Table 3. There was no indication of contamination between UC-Plus and VERSE treatment arms in the videos using the treatment fidelity monitoring form (Supplement 1). No UC-Plus treatment session included the key VERSE therapeutic elements.

**Table 3:** Therapy adherence by session in VERSE arm (*n*=252)

Adherence measure	Number of sessions adherent
Appropriate taska	237 (94.0%)
Therapy embedded in conversation^a^	236 (93.7%)
Appropriate task instructions^a^	233 (92.5%)
Appropriate cueing^a^	230 (91.2%)
Predominantly verbal task^a^	230 (91.2%)
Appropriate timing of cues^a^	227 (90.1%)
45-60 min direct intervention logged on REDCap^a^	221 (87.7%)
Target achieved in 3-4 attempts^a^	213 (84.5%)

^a^Key VERSE therapeutic elements

## Discussion

Post-stroke aphasia treatment fidelity in clinical trials has been commonly reported as ‘planned’, but lacks comprehensiveness for actual treatment fidelity outcomes reported [11]. We demonstrated that detailed treatment fidelity data collection and reporting against recommended frameworks is possible in a large-scale aphasia randomised control trial, and that fidelity reporting and analysis facilitated interpretation of how closely the planned intervention was delivered.

The relatively low rates of protocol deviation in VERSE give confidence that overall, the treatment(s) we intended to test were realised in this trial. We reported protocol and therapy deviations separately (see Supplement 2 for protocol deviations) as they represent different elements of the trial design. Therapy deviations were classified as deviations in the specific delivery of the intervention. Whilst 431 therapy deviations may seem a large number, it is important to note that with a trial of this size (3018 UC-Plus and VERSE service events) these deviations represent 1.4% of the overall services. Nevertheless, we do not know the threshold of acceptable deviations for efficacy or whether the number of deviations in our study is similar to other aphasia RCTs. We note the significant but small contribution of multiple patient, stroke and aphasia factors contributing to the number of therapy deviations in this study. This is likely to represent ‘real-life’ reasons for therapy not going ahead as per the planned protocol. These factors (increasing age, higher stroke and aphasia severity) are not surprising when considering their role in early stroke recovery. The fact that more therapy deviations occurred in the UC-Plus group may be related to patient-specific issues and or staffing-related issues. Our data were not coded to determine site-specific therapy deviations, in that all deviation data were linked via the original participant number which recorded the de-identified recruiting site only. The trial participant number did not reflect transfer of participants between healthcare sites. This point may be of use to the planning of future treatment fidelity studies.

We believe the reporting of all intervention protocol deviations is essential to provide deeper understanding of trial results. When planning the VERSE trial, we used pilot data from our previous research regarding challenges faced concerning dose and type of treatment delivery, to help design treatment fidelity processes in VERSE. A central tenant of treatment fidelity reporting is that it should be based on factual observation of the treatment, and not simply whether treatments were planned [5, 6, 11]. Given the multifactorial nature of complex interventions in aphasia trials, a level of deviation from the treatment plan is anticipated. How much deviation from intervention protocol constitutes a complete break from the original therapy intent is unknown.

Our main method of direct observation of the intervention was the use of video-recorded therapy sessions. Two-thirds of the expected video-recorded sessions were received (66%). We argue that this number of recordings is reasonable and a valid representation of the intensive intervention in the trial, given (a) there were over 240 therapists providing intensive treatment, (b) not every participant received every prescribed session and (c) significant technological challenges of involved in transferring large confidential data files. The VERSE intervention group therapists submitted more recordings than the UC-Plus therapists which may have been due to additional training received by these therapists. Four hundred thirty-seven videos (14.5% of the total number of treatment sessions) in the intensive arms of the trial were reviewed in their entirety for treatment fidelity, reflecting a logistically feasible number given the size of the VERSE trial. The issue of resourcing therapy monitoring in any study is significant and needs to be considered in the overall funding of intervention studies.

There is no accepted minimum level of integrity required in a complex, behavioural RCT; however, the literature suggests protocol adherence of 80% or greater to be considered a high-fidelity level [6, 7, 20]. An important finding from our study is the 98% protocol adherence to the VERSE treatment a priori defined key intervention ingredients, consistent with high protocol adherence in another study [13]. The VERSE treatment group reported less than half the number of therapy deviations compared to UC-Plus (137 compared to 294, respectively). This is likely due to the detailed and prescribed VERSE therapy regimen, facilitating adherence. Overall, these data indicate the higher intensity, clearly defined VERSE intervention was delivered as planned with few deviations, and treatment types between the different arms were clearly differentiated. Results can therefore be interpreted with confidence.

In complex behavioural interventions, the multiple planned elements of the intervention are interconnected, such that isolating and describing the therapeutic elements of a complex behavioural intervention is challenging. While study design and treatment tasks are frequently reported in aphasia trials, the theoretical underpinnings and potential active ingredients within the intervention are reported much less often [9, 11]. When delivering and evaluating an intervention such as aphasia therapy, conceptualising and developing the therapeutic elements into a measurable protocol can seem overwhelming. It is therefore not surprising that measuring protocol adherence in aphasia intervention is poorly executed. The proposed macrostructure for measuring treatment fidelity, outlined by TIDieR [3] and Bellg [5], that reinforces the use of a measurable theoretical approach, the mapping of this to planned treatment tasks, and measuring the implementation of the overall study protocol, offers a robust guide for including this critical dimension of intervention research.

We note gaps in reporting requirements between CONSORT [4], Standard Protocol Items: Recommendations for Intervention Trials SPIRIT [21], TIDieR [3] and Bellg et al. [5] related to treatment fidelity. The CONSORT and SPIRIT statements provide a broad overview of intervention reporting standards, designed to be supported by the TIDieR statement. To report a level of detail that allows true replication of a complex behavioural intervention, we found the BCC framework an essential addition to reporting aphasia treatment fidelity.

### Study limitations

Limitations of the reporting of treatment fidelity in this trial related to limited video recordings in the UC arm. Whilst it was mandatory for therapists in the intensive arms of the trial to video-record sessions, the UC therapists were only encouraged to record sessions, and only two sessions were received, one of which was corrupted. Therefore, limited data was available for UC [17].The addition of treatment fidelity data for the UC arm would have provided valuable information about therapy adherence and differentiation between all therapy arms in the trial. The 66% of expected videos received were assumed to be representative of treatment as a whole; however, the possibility exists that it was not. The Hawthorne effect [18] may play an undetermined role whereby therapists changed their behaviour once aware they were being recorded. We believe this to be an unavoidable bias in the reporting of fidelity currently. Further, the treatment fidelity reported here was completed at the macrostructure level. Previously published work [22, 23] presents finer grained, utterance level analyses and adds nuanced therapeutic information to the efficacy picture. Finally, the receipt of treatment and enactment of treatment skills areas of Bellg et al. [5] were not measured in this study and so cannot be commented on.

### Future directions

A main finding of this study indicated greater treatment protocol adherence in the arm of the trial that received the highest protocol training and more detailed treatment resources (including manualised treatment plans and within treatment session reporting). This suggests that greater detail in a treatment protocol coupled with increased training may result in increased protocol adherence. We hope that future studies will incorporate fidelity treatment processes similar to those outlined to build robust reporting systems for aphasia research to increase and improve reporting of treatment fidelity. The detailed level of treatment reporting is not exclusive to randomised controlled trials and should be reflected in all treatment research with this population. The critical nature of full treatment fidelity reporting when interpreting evidence highlights the importance of this level of detailed planning in aphasia research. The importance of explicit reporting of the theoretical rationale and the potential active ingredients as part of the study design is stressed here as the first step to measuring treatment fidelity. This will promote identification and subsequent measurement of hypothesised therapeutic elements, enabling their examination when assessing the fidelity of the study treatment being evaluated. Exploring ways to conceptualise and measure treatment enactment is an area of need in future research. Investigating the measurement of trained-skills carryover to naturalistic settings presents a significant challenge to aphasia intervention design and evaluation and inches us forward in the measurement of treatment efficacy.

## Conclusion

We devoted substantial financial, logistical and intellectual resources to treatment fidelity, heeding comments from the BCC. The overall value of the detailed fidelity reporting in this clinical trial allows for strengthened interpretation of the ‘null’ VERSE trial results, in that, the early aphasia recovery does not appear to be enhanced by an intensive therapy regimen. Further research is urgently required to determine dose-related responses to treatment in early recovery. Commencing aphasia effectiveness research with a detailed treatment fidelity plan and reporting structure is essential. Vigilant monitoring and reporting of all intervention components will only enhance the aphasia research discovery pipeline [24].

## Supplementary Information

**Additional file 1.** Therapy integrity monitoring form.

**Additional file 2.** VERSE Prescribed treatment arm goal levels.

**Additional file 3.** Study protocol deviations. Table S3. Study protocol—assessment deviations by treatment arm.

## Data Availability

The datasets used and/or analysed during the current study are available from the corresponding author on reasonable request.

## Abbreviations

VERSE: Very Early Rehabilitation in SpEech
RCT: Randomised controlled trial
UC: Usual Care
UC-Plus: Usual Care-Plus
TIDieR: Template for Intervention Description and Replication
CONSORT: Consolidated Standards of Reporting Trials
BCC: Behaviour Change Consortium
AQ: Aphasia Quotient
SPIRIT: Standard Protocol Items: Recommendations for Intervention Trials
