# Supplementary figures and images for: Treatment fidelity monitoring, reporting and findings in a complex aphasia intervention trial: a substudy of the Very Early Rehabilitation in SpEech (VERSE) trial
Source: Trials. 2022 Jun 16;23:501. doi: 10.1186/s13063-022-06433-3 (PMC9204960; doi:10.1186/s13063-022-06433-3)

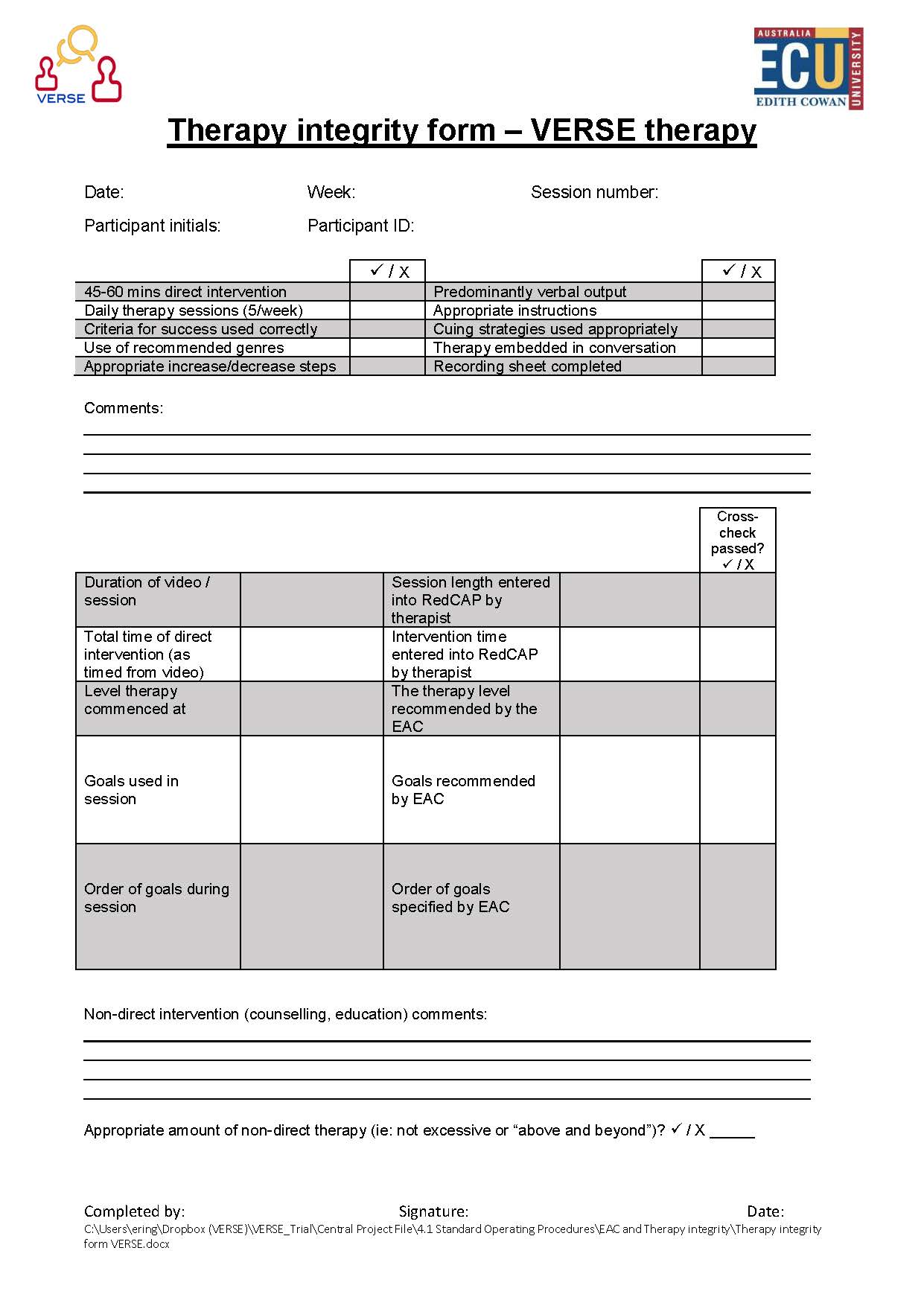


Supplement 1. Therapy integrity monitoring form

Supplement: Supplementary file 1 — Additional file 1. Therapy integrity monitoring form. [file 13063_2022_6433_MOESM1_ESM.docx]
