# Supplementary material for: Treatment fidelity monitoring, reporting and findings in a complex aphasia intervention trial: a substudy of the Very Early Rehabilitation in SpEech (VERSE) trial
Source: Trials. 2022 Jun 16;23:501. doi: 10.1186/s13063-022-06433-3 (PMC9204960; doi:10.1186/s13063-022-06433-3)
Supplement: Supplementary file 2 — Additional file 2. VERSE Prescribed treatment arm goal levels. [file 13063_2022_6433_MOESM2_ESM.docx]

| Goal level | Definition |
| --- | --- |
| 1a | Receptive: Identification of verb pictures from spoken words |
| 1b | Receptive: Identification of noun pictures from spoken words |
| 1c | Receptive: Identification of adjectives pictures from spoken words |
| 2 | Verbal production of single words |
| 3 | Verbal production of two element phrases or clauses |
| 4 | Verbal production of three element phrases or clauses |
| 5 | Verbal production of complex clauses and/ or phrases |
| 6 | Verbal production of complex phrases (verb and noun) and clauses |
| 7 | Verbal conversation about familiar topics |
| 8 | Verbal conversation about unfamiliar topics |

Supplement 2 VERSE Prescribed treatment arm goal levels
