# Supplementary material for: Treatment fidelity monitoring, reporting and findings in a complex aphasia intervention trial: a substudy of the Very Early Rehabilitation in SpEech (VERSE) trial
Source: Trials. 2022 Jun 16;23:501. doi: 10.1186/s13063-022-06433-3 (PMC9204960; doi:10.1186/s13063-022-06433-3)
Supplement: Supplementary file 3 — Additional file 3. Study protocol deviations. Table S3. Study protocol—assessment deviations by treatment arm. [file 13063_2022_6433_MOESM3_ESM.docx]

Supplement 3

### Study Protocol deviations

A protocol deviation was defined as a departure from the main study protocol^17^. A total of 410 study protocol deviations were logged in the REDCap® system. The quantity and reasoning behind the deviations are presented in Table 3.

Table 3. Study protocol – Assessment deviations by treatment arm

|  | UC (n=183) | UC-Plus (n=124) | VERSE (n=103) |
| --- | --- | --- | --- |
| Assessment not performed |  |  |  |
| Fatigue | 13 (7.1%) | 12 (9.7%) | 10 (9.7%) |
| Medically unwell | 4 (2.2%) | 5 (4.0%) | 4 (3.9%) |
| Refusal | 8 (4.4%) | 6 (4.8%) | 4 (3.9%) |
| Deceased | 7 (3.8%) | 5 (4.0%) | 0 (0%) |
| Other | 9 (4.9%) | 0 (0%) | 0 (0%) |
| Assessment performed but incomplete | 78 (42.62%) | 69 (55.6%) | 21 (20.4%) |
| Week 12 assessment late^ | 19 (10.4%) | 8 (6.5%) | 23 (22.3%) |
| Week 26 assessment late^ | 18 (9.8%) | 14 (11.3%) | 13 (12.6%) |
| Other protocol deviation* | 27 (14.8%) | 5 (4.0%) | 28 (27.2%) |

^ outside the protocol period of 7 days; *A text response box was provided in REDCap and reasons given were highly varied.
